# Bioinformatics prediction of overlapping frameshifted translation products in mammalian transcripts

**DOI:** 10.1186/1471-2164-9-122

**Published:** 2008-03-06

**Authors:** Sebastien Ribrioux, Adrian Brüngger, Birgit Baumgarten, Klaus Seuwen, Markus R John

**Affiliations:** 1 Genedata AG, Maulbeerstrasse 46, CH-4016 Basel, Switzerland; 2 Basilea Pharmaceutica AG, Grenzacherstrasse 487, CH-4005 Basel, Switzerland; 3 Novartis Institutes for Biomedical Research, CH-4002 Basel, Switzerland

## Abstract

**Background:**

Exceptionally, a single nucleotide sequence can be translated *in vivo *in two different frames to yield distinct proteins. In the case of the G-protein alpha subunit XL-alpha-s transcript, a frameshifted open reading frame (ORF) in exon 1 is translated to yield a structurally distinct protein called Alex, which plays a role in platelet aggregation and neurological processes. We carried out a novel bioinformatics screen for other possible dual-frame translated sequences, based on comparative genomics.

**Results:**

Our method searched human, mouse and rat transcripts in frames +1 and -1 for ORFs which are unusually well conserved at the amino acid level. We name these conserved frameshifted overlapping ORFs 'matreshkas' to reflect their nested character. Select findings of our analysis revealed that the G-protein coupled receptor GPR27 is entirely contained within a frame -1 matreshka, thrombopoietin contains a matreshka which spans ~70% of its length, platelet glycoprotein IIIa (ITGB3) contains a matreshka with the predicted characteristics of a secreted peptide hormone, while the potassium channel KCNK12 contains a matreshka spanning >400 amino acids.

**Conclusion:**

Although the *in vivo *existence of translated matreshkas has not been experimentally verified, this genome-wide analysis provides strong evidence that substantial overlapping coding sequences exist in a number of human and rodent transcripts.

## Background

Overlapping translated open reading frames (tORFs) are usually associated with genomes under selection pressure to remain compact, such as those of viruses. However, such overlaps also exist in mammals. For example in human, an exon is shared by the INK4A and ARF genes and is translated in different frames over 317 bases [1]. Similarly, a transcript fusion between the human EIF4EBP3 and MASK genes results in the translation of 172 bases in two different frames [2]. An alternative splice variant of insulin-like growth factor 1 (IGF-I), called mechano-growth-factor (MGF), contains a frameshift which leads to translation of overlapping reading frames [3]. Expression profiling of IGF-1 and MGF indicates that the variants have distinct physiological roles.

**Figure 1:**
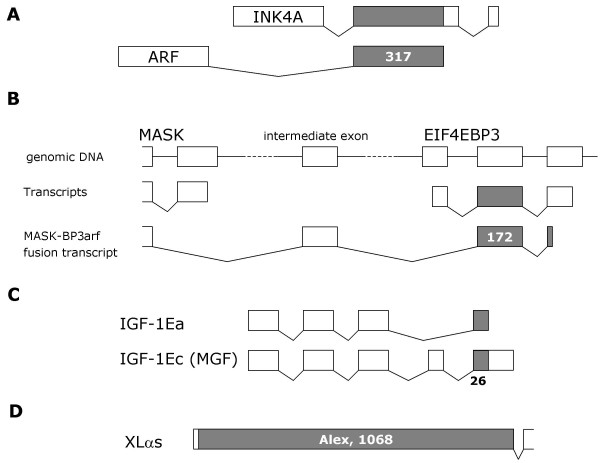
**Known examples of overlapping translated ORFs**. A) The second exon of the INK4A and ARF genes is shared but translated in different reading frames. B) A transcript fusion can occur between the MASK and EIF4EBP3 genes, via an intermediate exon. In this case, the next exon is translated in a different frame from that in EIF4EBP3 transcripts. C) A splice variation in IGF-1 can cause a frameshift in the translation of C-terminal residues. D) Exon 1 of the G-protein subunit XL-alpha-s contains a second, frameshifted tRF which yields a distinct protein called Alex. Grey shading indicates overlapping frameshifted ORFs, exons and tORFs are drawn approximately to scale.

The best-characterised case of overlapping tORFs in mammals is that of XL-alpha-s. This is a splice variant of a G protein alpha subunit, derived from the GNAS complex locus, which is expressed in neuroendocrine tissues and other tissues. The first exon of XL-alpha-s contains a downstream ORF which is frameshifted +1 relative to the XL-alpha-s initiator codon. This ORF gives rise to an entirely different protein called Alex, which is 356 amino acids long in rat [4]. Remarkably, XL-alpha-s and Alex interact, and this interaction can be disrupted by an insertion polymorphism in humans. The polymorphism leads to enhanced receptor-mediated cAMP formation in platelets and fibroblasts, increased trauma-related bleeding tendency, and in two families neurological problems and brachydactyly were observed [5]. Furthermore, the XL-alpha-s and Alex ORFs may extend in the 5' direction for several hundred nucleotides more [6], raising the possibility that longer variants of XL-alpha-s and Alex exist. Although the Alex termination codon lies well within 50 base pairs (bp) of the next 3' splice junction, the XL-alpha-s transcript does not appear to be degraded according to the usual rules for nonsense-mediated decay [7]. Figure 1 summarises these cases of overlapping mammalian tORFs.

During our own *in silico *comparative studies of entire translated human, mouse and rat genomes, we frequently observed overlapping tORFs conserved at the amino acid level. In an effort to explore this relatively uncharacterised aspect of gene structure and evolution, we screened for additional Alex-like cases in human and rodents using a bioinformatics approach. Specifically, we searched human, mouse and rat transcripts for frameshifted conserved tORFs. Conservation of such sequences at the amino acid level may reflect a functional role. A related comparative genomics approach, supported by simulation-based statistics, has recently been published [8]. Based on conservation between human and mouse, Chung *et al. *convincingly demonstrate that these frameshifted ORFs (which they name alternative reading frames, or ARFs) are highly unlikely to occur by chance. In our study, the term 'matreshka' was coined to describe the overlapping tORFs, in analogy with Russian dolls, as one protein can be thought of as 'hiding' another. It should be kept in mind, however, that all matreshkas reported here are observations based on mRNA sequence. Translation of these sequences *in vivo *has not yet been experimentally confirmed.

## Results and Discussion

**Figure 2:**
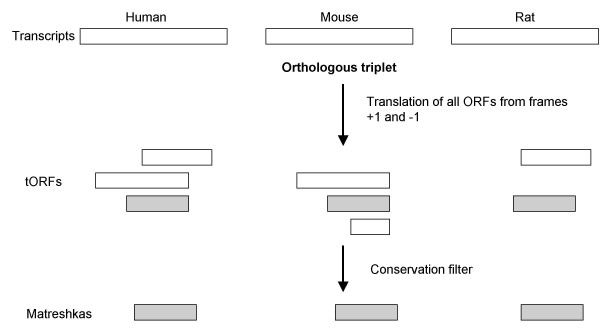
**Matreshka bioinformatics pipeline**. A) Human, rat and mouse transcripts were assembled into orthologous triplets, and all frame +1 and -1 ORFs greater than 50aa in length translated *in silico *(frames +1 and -1 are sometimes also known as frames 2 and 3, respectively). A conservation filter was then applied to yield matreshka predictions.

Matreshkas are defined here as overlapping, frameshifted ORFs (relative to a known ORF) in transcripts, which are well conserved at the amino acid level. Matreshkas may potentially represent alternative translation products like Alex, or suggest the existence of functional frameshifted splice variants. To obtain matreshka predictions, we translated *in silico *all frame +1 and -1 ORFs from known human, mouse and rat "parent" transcripts, and applied a conservation filter (Fig. 2).

### Open reading frame translation

The first step was to build Human-mouse-rat ortholog triplets, from which frame +1 and -1 ORFs were extracted (frames +1 and -1 are also sometimes known as frames 2 and 3, respectively). Ortholog sets were based on a variation of best reciprocal BLASTP hits, which favoured hits with a higher percentage identity (see Methods). This method yielded 9163 triplets. The coding nucleotide sequence was retrieved for each ortholog, and all translated ORFs (tORFs) at least 50 amino acids (aa) long were extracted computationally.

The matreshka identification pipeline was run twice, each time varying the tORF extraction method. In an effort to identify Alex-like cases, the first run retained only tORFs beginning with methionine (labelled here 'tORF_M_'). The second run was somewhat more comprehensive, including translated ORFs starting with any amino acid (labelled 'tORF_X_'). It should be kept in mind that although the tORF_M _set is contained within the tORF_X _set, the derived matreshka sets only partially overlap, due to the properties of the conservation filter. The term 'tORF' is used here to collectively refer to tORF_M _and tORF_X_.

**Figure 3:**
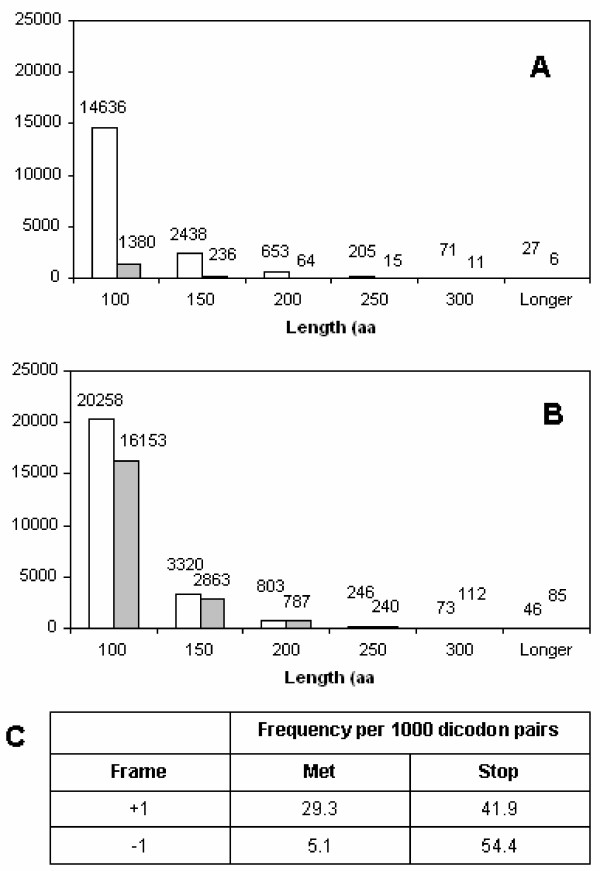
**Length distribution of human tORFs, broken down by frame**. A) redundant methionine-start translated ORFs (tORF_M_) and B) non-redundant tORFs beginning with any amino acid (tORF_X_). White bars correspond to frame +1 tORFs, grey bars correspond to frame -1. C) Frequencies of methionine and stop codons in frames +1 and -1.

This early stage in the analysis, after tORF extraction but before the conservation filter, already yielded surprising results: tORFs beginning with methionine (tORF_M_) were found to be ~10-fold rarer in frame -1 than in frame +1 (Fig. 3A). This effect was also seen for tORF_x_, but in a much less pronounced way (Fig. 3B). The pattern was mirrored by human codon usage frequencies (Fig. 3C): stop codons are more frequent in frame -1 than frame +1, resulting in fewer frame -1 tORFs. In addition, ATG codons are ~5-fold rarer in frame -1 compared to +1, accounting further for the observed scarcity of frame -1 tORF_M_. In the ARF study [8], the difference between the two frames was echoed in simulated alignments. The importance of this apparent suppression of tORF_M _may be part of a mechanism to minimize the impact of translation initiation errors. Why there should be a difference between frames +1 and -1, however, is unclear. Perhaps an alternative mechanism already exists to prevent erroneous frame +1 translation initiation.

Examination of amino acid sequences in the parent frame sheds some light on how stop codons can occur in other frames. If, for instance, a tyrosine (codons TAT or TAC) is followed by an aspartic acid (codons GAT or GAC), the stop codon TGA occurs in frame -1 if, and only if, the codon used for tyrosine is TAT. Overall, codon TAT is used for tyrosine in 45% of the cases in human genes. However, when tyrosine is directly followed by aspartic acid in a coding sequence, TAT occurs in 75% of the cases to code for tyrosine (Table 1). Thus, the codon which leads to a stop codon in frame -1 is favoured.

**Table 1:** Bi-codon frequencies. The third column lists those codons c for the first amino acid (AA1) in a consecutive pair which lead to a stop in frame -1. The overall relative frequency of c as a codon for AA1 is given in the fifth column. The relative frequency of c in the specific context AA1-AA2 is given in column six. The penultimate column shows the enrichment of the codon c in this context; an enrichment > 1 indicates that this codon is more frequently used for AA1 when it leads to a stop in frame -1 than in the average case. Probability (P) values were assigned using the χ^2 ^test, and significance determined with a Bonferroni multiple testing correction (15 analyses). Single (*) and triple (***) asterisks indicate significance at the 0.05 and 0.001 levels, respectively.

**AA1**	**AA2**	**First codon leading to stop in frame -1**	**Overall frequency**	**Frequency when leading to stop**	**Enrichment**	**P**
A	D	GCT	27	38	1.4	0.034
C	D	TGT	45	80	1.8	1.814e-07 ***
D	D	GAT	46	78	1.7	2.38e-06 ***
F	D	TTT	46	80	1.7	5.358e-07 ***
G	D	GGT	16	23	1.4	0.08
H	D	CAT	42	69	1.6	3.097e-05 ***
I	D	ATT	36	55	1.5	0.002 *
L	D	CTT	13	12	0.9	0.782
N	D	AAT	47	78	1.7	6.131e-06 ***
P	D	CCT	29	36	1.2	0.194
R	D	CGT	8	9	1.1	0.724
S	D	TCT, AGT	34	51	1.5	0.004 *
T	D	ACT	25	31	1.2	0.230
V	D	GTT	18	19	1.1	0.814
Y	D	TAT	44	75	1.7	2.962e-06 ***
W	D	-	-	-	-	
Q	D	-	-	-	-	
M	D	-	-	-	-	
K	D	-	-	-	-	
E	D	-	-	-	-	

In order to explore whether this observation can be generalized, we calculated a 64 by 64 bi-codon usage matrix for a set of human coding sequences (see Additional file 1). Table 1 shows as an example the combinations of two consecutive amino acids (bi-codons), where the second amino acid is aspartic acid (both codons for aspartate begin with AG, therefore the occurrence of a stop codon in frame -1 depends entirely on the first codon). Interestingly, there is a trend for codons to be favored which lead to a stop codon in the frame -1, in some cases leading to highly significant probability values (Table 1). The same effect can be observed when the second amino acid in the bi-codon is glutamic acid (Additional file 1). In contrast, if the second amino acid is lysine or asparagine, the opposite trend appears (Additional file 1). This may be due to interacting effects on the complementary nucleotide strand, and illustrates the complexity of bi-codon effects on the appearance of stop codons in frame -1. Note that our analysis did not take into account the base following the stop codon, which can have a significant effect on efficiency of termination of translation [9].

### Conservation filter

Having extracted all tORFs, the conservation filter was then applied. Within each ortholog triplet, all human frame +1 and -1 tORFs were compared in pairwise global alignments to all those computed from mouse and from rat. Well-conserved tORF triplets which passed the length similarity and identity criteria were retained for further analysis as candidate matreshkas. Matreshkas derived from tORF_M _are denoted here as matreshka_M_, those derived from tORF_X _are denoted matreshka_X_, while a lack of subscript refers to both matreshka sets collectively. The breakdown of tORF and matreshka statistics by frame is given in Table 2.

**Table 2:** Matreshka statistics. Matreshka statistics for tORFs and met-tORFs (generated from a separate run of the analysis, see text), broken down by frame. Note that tORF_M _are redundant (see Methods).

**# Human-Mouse-Rat ortholog triplets**	9163			
	**Frame +1**	**Frame -1**	**Total**	**Frame -1 as a percentage of total**

**# tORF**_X_	24 746	20 240	44 986	45%
**# matreshka**_X_	5 764	1 915	7679	25%
**# tORF**_M_	18 030	1 711	19 741	9%
**# matreshka**_M_	1 689	104	1793	6%

Of the 7679 matreshka_X _(beginning with any amino acid), 1853 are at least partially redundant with the matreshka_M _(beginning with methionine only), of which there are 1793 in total. The matreshka_M _total (1793) is smaller than the number of redundant matreshka_X _(1853), because the longer matreshka_X _can contain several of the shorter matreshka_M_. Frame -1 tORFs were filtered out more effectively than frame +1 (Table 2): the proportion of frame -1 tORF_X _dropped from 45% to 25% of the total after conservation filtering, and from 9% to 6% for tORF_M_. The greater stringency of the conservation filter on frame -1 tORFs was expected, and is a consequence of the variation in the third base of each parent codon: this can be demonstrated in a simple exercise by combining all possible nucleotide triplet pairs. For a given amino acid in frame 0, on average ~5 different amino acids can potentially be coded for in frame +1, while ~15 can be coded for in frame -1 (data not shown). The scarcity of frame -1 tORF_M_, combined with the greater efficacy of the conservation filter, resulted in very few (104) matreshka_M _in frame -1. For reference, Alex lies in frame +1.

No matreshkas were found corresponding to Alex/XL-alpha-s [4], INK4A/ARF [1] or 4E-BP3/MASK [2] because they were not represented in the ortholog triplets (primarily because a RefSeq sequence was not available for all three organisms, human, mouse and rat) and in the case of IGF1 [3] because the region which is translated in two frames is short (16aa, the matreshka length filter being 50aa). As recovery of Alex-like entities but not Alex itself was our primary goal, we used stringent conservation filters that Alex could not have passed: human Alex is only 53% and 55% identical at the amino acid level to mouse and rat Alex, respectively [4], while our conservation threshold was set at 60%. This relatively high threshold was necessary partly because the exploratory nature of the project made clear-cut examples necessary, but was also based on separate benchmark studies we made on the chemokine family. Chemokines are typically quite poorly conserved, and as such provide an indication of how much sequences may diverge while retaining similar functions. For instance, half of mouse-human chemokine orthologs in Homologene [10,11] have an amino acid sequence identity of 60–70% (from 32 entries in total). Since a 60% cut-off seems suitable for this family, and we were interested in detecting possible new peptide ligands, it was chosen as a threshold for the matreshkas.

A number of matreshkas mapped to alternative, frameshifted splice variants (see Additional file 2). In some cases, there is evidence that these variants are functionally distinct. Among these are the paired box gene 8 (PAX8) isoform c [12], and X-box protein (XBP1) isoform U [13].

Given the nucleotide sequence similarity between transcripts in orthologous triplets, conserved translation products in alternative reading frames would be expected to occur by chance. Additional parameters were required to differentiate likely matreshkas from false positives. Generally, longer matreshkas are less likely to arise by chance, therefore matreshka length was chosen as a first parameter for selection. A second useful parameter gave an approximate measure of the selection pressure maintaining a potential matreshka: this was the number of amino acid positions where a stop codon could have arisen, truncating the matreshka but leaving the parent (RefSeq) protein sequence unaffected (see Methods for an explicit example), denoted here N_stop_. Furthermore, we estimated a probability of seeing N_stop _given the parent amino acid sequence, and considering codon bias (see Methods), but not taking into account the effects of bi-codon bias mentioned above (see 'Open reading frame translation' section).

To gain clues as to the possible function of matreshkas, all matreshka protein sequences were analysed with functional motif and signal peptide prediction programs. Matreshka nucleotide sequences were also screened for tandem repeats, to exclude artifactual sequences which pass the length filter (e.g., if the repeat contains no stop codon in the matreshka frame). Matreshkas with tandem repeats in any one of the three organisms were discarded from in-depth analysis, even though Alex itself was found to contain such repeats. In addition, matreshka_M _nucleotide sequences were scanned for consensus Kozak translation initiation motifs [14,15], to help determine whether leaky ribosomal scanning [16] may be occuring. To estimate how frequently Kozak motifs appear by chance, we scanned the 9163 human coding sequences used for building matreshkas, and counted how frequently ATG occurred versus full Kozak sequences (all frames included). On average, ~30 ATGs, of which ~5 (16%) were Kozak motifs, were observed per transcript. Of the 1793 matreshka_M_, only 217 (12%) have a Kozak motif exactly at the start codon (data in Additional file 3). However, of the 15 longest matreshka_M_, 8 have a Kozak motif exactly at the start codon, which is a significant enrichment of true positives (exact binomial test, N = 15, P = 0.0001).

**Figure 4:**
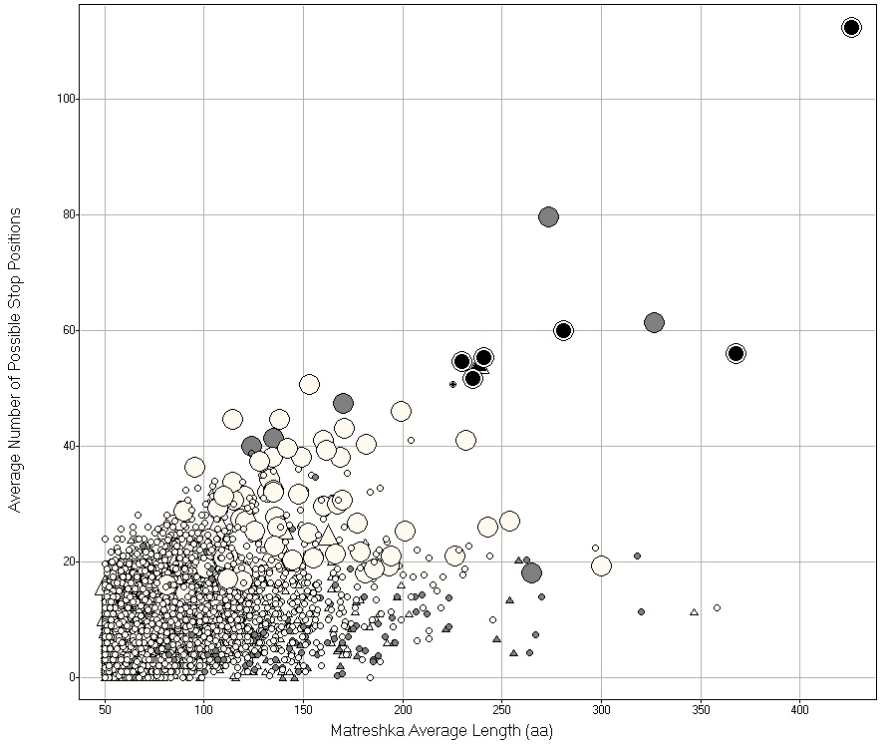
**Matreshka distribution**. Five parameters are displayed: matreshka amino acid length (x-axis), the number of positions where a stop codon could have interrupted the matreshka (y-axis), presence of tandem repeats (matreshkas with no measured tandem repeats in any species are in white, matreshkas with tandem repeats in any species are in grey), whether the matreshka starts with any amino acid (circles) or with methionine only (triangles), and whether the probability of matreshka truncation by a stop codon is lower (small shapes) or higher (large shapes) than 0.999. The most interesting candidates are the longest matreshkas with the highest number of possible stop positions, and with no measured tandem repeats (black).

The matreshka N_stop _versus length distribution can be seen in Fig. 4, with outliers of interest highlighted (data available in Additional file 3). Details on the highlighted matreshkas can be found in Table 3. Some matreshkas can be extended into 5' or 3' untranslated regions (UTRs) or (in the case of KCNK12 or POLG) into flanking genomic sequence (in total, 365 matreshkas extend 5' past the boundary of the parent RefSeq). Of particular interest is the THPO matreshka, which is of the rare methionine-start, frame -1 variety.

**Table 3:** Characteristics of selected matreshkas. Characteristics of the selected matreshkas from Fig. 4, for human (*H.s.*), mouse (*M.m.*) and rat (*R.n.*). Columns from left to right detail the database accessions of RefSeq transcripts used to extract matreshkas, the RefSeq description, the frame the matreshka lies in, the length in amino acids of the matreshka, whether the matreshkas start with methionine, and the number of positions where a stop codon could have occurred to interrupt the matreshka but leave the RefSeq parent unaffected.

**RefSeq parent accessions (*H.s.*, *M.m.*, *R.n*)**	**Parent description**	**Frame**	**Matreshka aa lengths (*H.s.*, *M.m.*, *R.n*)**	**Methionine Start**	**Number of possible stop positions in matreshkas (*H.s.*, *M.m.*, *R.n*)**
NM_022055; NM_199251; NM_022292	potassium channel, subfamily K, member 12 (KCNK12)	-1	418; 430; 430	No	108; 114; 115
NM_018971; NM_008158; NM_023099	Superconserved receptor expressed in brain 1 (GPR27)	-1	375; 356; 372	No	60; 53; 55
NM_022571; NM_181752; NM_181771	G protein-coupled receptor 135 (GPR135)	-1	290; 284; 268	No	59; 64; 57
NM_000460; NM_009379; NM_031133	thrombopoietin (THPO)	-1	255; 230; 230	Yes	55; 56; 52
NM_002693; NM_017462; NM_053528	polymerase (DNA directed), gamma (POLG)	-1	241; 224; 224	No	53; 55; 56
NM_153675; NM_010446; NM_012743	forkhead box A2 (FOXA2)	-1	236; 256; 213	No	52; 58; 45
NM_139315; NM_009315; XM_213729	TAF6 RNA polymerase II (TAF6)	-1	235; 220; 220	No	53; 50; 49
NM_000212; NM_016780; NM_153720	platelet glycoprotein IIIa (ITGB3)	+1	192; 192; 192	Yes	6; 6; 6

The matreshkas in Table 3 which don't begin with methionine are unlikely to represent complete proteins, since the human matreshka sequence either contains no methionines (KCNK12, GPR135, POLG, FOXA2) or methionines only very close to the 3' end of the matreshka (GPR27, TAF6). As with Alex and virtually all matreshka predictions, none of the candidates in Table 3 contain known protein functional motifs, as ascertained by pattern matching to Prosite motifs. The following sections describe matreshkas of particular interest.

### Case study 1: Matreshka framing super conserved receptor expressed in brain 1 (GPR27)

**Figure 5:**
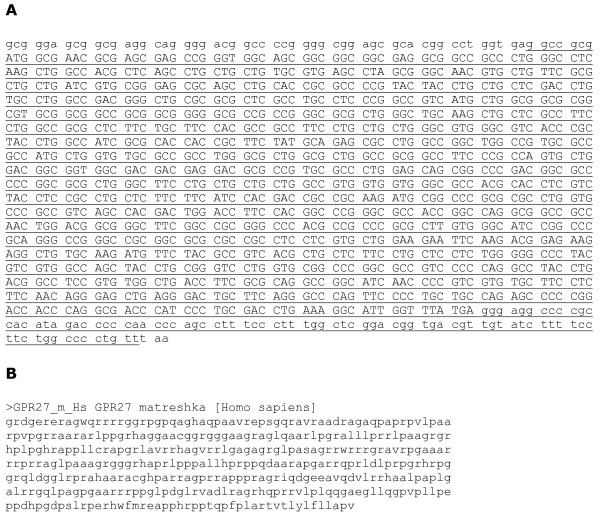
**G protein-coupled receptor 27 matreshka**. A) mRNA sequence of human GPR27 with matreshka underlined. GPR27 codons are separated by spaces, and flanking non-GPR27-coding-sequence is shown in lower case. B) human matreshka protein sequence.

The first example presented here is of a particularly large matreshka associated with the GPR27 transcript, also called super conserved receptor expressed in brain 1. GPR27 is an aminergic G-protein coupled receptor, cloned from human brain cDNA and part of a family of three members which are highly conserved between vertebrates [17]. This single-exon coding sequence is entirely contained within a frame -1 matreshka_X_, which begins and ends in the flanking UTRs (Fig. 5A). The matreshka_X _varies in length between 407 and 418 amino acids depending on the species, and in the RefSeq coding region alone has between 53 (mouse) and 60 (human) positions where a stop codon could have truncated the matreshka_X_, suggesting selection pressure to maintain it. the probability in human of a stop codon truncating the matreshka, P_stop_, was estimated at 0.999997. It seems possible from our analysis that the high level of conservation of this gene may be necessary to maintain the protein sequences of both GPR27 and the matreshka. Figure 5B shows the matreshka_X _amino acid sequence, which like Alex is basic (predicted pI 12.9) and enriched in proline residues (~3-fold more than average). The GPR27 matreshka is unlikely to be translated from a leaky scanning event [16], since it contains only one methionine residue 30 amino acids from its C-terminus. It may be part of the exon of a longer, unknown transcript, in which case the AG straddling its 5' end could be a canonical splice signal.

### Case study 2: Matreshka contained in thrombopietin (THPO)

**Figure 6:**
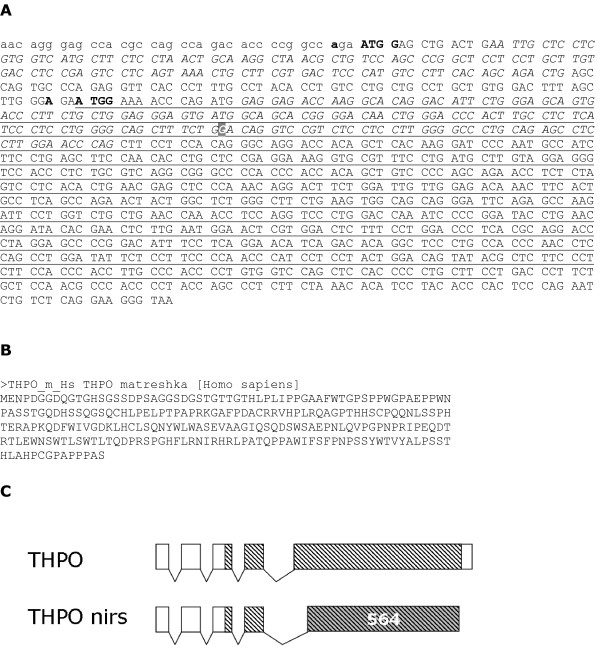
**Thrombopoeitin matreshka**. A) mRNA sequence of thrombopoietin (THPO), with matreshka underlined. 5'UTR sequence is shown in lowercase, Kozak motifs in bold. The bold white guanine base (grey background) can be replaced by adenine to truncate the matreshka through a SNP (NCBI ID rs1126665), and replaces a glycine with a glutamate within the Erythropoietin-Thrombopoietin motif (Pfam ID PF00758.7). Alternating italics/non-italics represent alternate THPO coding exons, and spaces separate codons for the parent RefSeq. B) matreshka protein sequence. C) Illustration of the translation of the THPO matreshka in the nirs splice variant. THPO splice variant 1 is shown above the nirs splice variant. Hatching indicates the location of the matreshka. Nirs splicing utilises an alternative acceptor which results in the last exon being translated in the same frame as the matreshka over 564 nucleotides (indicated by grey shading).

Thrombopoietin is the major regulator of platelet production by megakaryocytes, with associated disorders including thrombocytosis and thrombocytopenia. Splice variant 1 of THPO contains a matreshka of the scarce frame -1 methionine-start type, which in human is 254 amino acids long, and covers 72% of the length of THPO (Fig. 6A). It contains 55 positions where a stop codon could have occurred without changing the parent RefSeq protein sequence (P_stop _= 0.999999). For comparison, in frame +1 there are 30 positions where a stop codon could occur, and 12 stop codons actually did. Like Alex, the THPO matreshka is proline-rich (Fig. 6B).

The genomic region upstream of the matreshka_M _start was computationally scanned for transcription start sites (TSS), in case it is in fact derived from a separate transcript overlapping with the THPO sequence: none was found. The matreshka_M _start is 212 nucleotides downstream from the parent start, and the initiator ATG lies in a strong Kozak context (Fig. 6A) in all three organisms. This suggests that the matreshka could be produced from a leaky ribosomal scanning event [16].

An important indication, however, that the THPO matreshka_M _may be translated not from a leaky scanning event, but instead as part of a frameshifted splice variant, came from a BLAST search of the matreshka_M _against the UniProt protein database [18]. A 100% identity match was found against the translation of the 'nirs' THPO splice variant. No function specific to the nirs THPO appears to be documented. This variant contains a frameshift downstream of the matreshka_M _start, which effectively places the 3' half of the transcript in the same frame as the matreshka_M _(Fig. 6C).

A SNP [19] exists which can generate a stop codon in (and thus truncate) the matreshka_M _at the 45th amino acid (Fig. 6A). The SNP replaces a glycine with a glutamate at position 116 in THPO itself, on a loop between an alpha-helix and a beta sheet. Gly^116 ^is within the conserved, 184aa long Erythropoietin-Thrombopoietin domain in the N-terminal portion of THPO. This domain is responsible for binding to THPO's receptor, MPL (myeloproliferative leukemia virus oncogene). Three independent site-directed mutagenesis studies have been carried out to determine which residues are necessary for binding to MPL, based on 3D structural data [20-22]. A total of 14 amino acids were determined to be important for binding, none of which lie within 15aa of Gly^116^. It therefore seems unlikely that the SNP affects THPO function. Also of note are two splice variants (labelled '2' and '3' in RefSeq), which retain the SNP and shorten the matreshka_M _by 4 and 39 aa, respectively.

### Case study III: Matreshka contained in Platelet glycoprotein IIIa (ITGB3)

**Figure 7:**
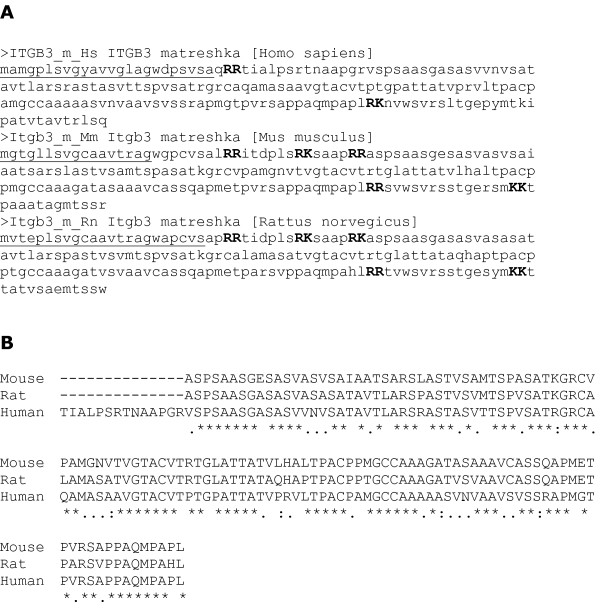
**Integrin beta 3 matreshka**. A) Amino acid sequences of the human, mouse and rat ITGB3 matreshkas, with predicted signal peptide underlined, and predicted dibasic cleavage sites in bold capitals. B) The largest fragment produced by cleavage at dibasic sites would be very well conserved, as shown by this global alignment.

Platelet glycoprotein IIIa (GPIIIa; also known as integrin beta 3, ITGB3) is part of the GPIIb/GPIIIa complex, which mediates platelet aggregation by acting as a receptor for fibrinogen. A frame +1 matreshka_M _(the same type as Alex) spans 3 exons in the ITGB3 transcript. It is 192 amino acids long in human, and has strong Kozak sequences and a signal peptide prediction in all three organisms (Fig. 7A), coupled with dibasic cleavage sites clustered at the N and C-termini which could potentially produce conserved 'active' secreted peptides (Fig. 7B). Such characteristics are typical of GPCR ligands, for example [23], which are of great value for drug discovery. From an evolutionary perspective, it seems plausible that such a secreted peptide could 'signal' the expression of ITGB3, since any putative receptor would be able to freely co-evolve with the matreshka, according to the sequence requirements of ITGB3 itself.

The significance of the matreshka's Kozak motif is underlined by the absence of a Kozak motif for the parent sequence: the first motif in the ITGB3 frame occurs 934 nucleotides downstream of the annotated start codon. This suggests that leaky ribosomal scanning is occurring in the parent frame, and may increase the likelihood that the matreshka_M _(a further 490 nucleotides downstream) is also translated from leaky scanning.

These are by no means the only interesting predictions. The largest matreshka discovered is 448aa long in human, although it has the potential to reach up to 500aa in mouse (Table 3). It is derived from the KCNK12 potassium channel, a member of the 2-pore domain superfamily of background K^+ ^channels. The G protein-coupled receptor 135, the gamma subunit of a DNA directed polymerase, TAF6 RNA polymerase II, and Forkhead box A2 also contain substantial size matreshkas (Table 3). These matreshkas may represent alternative proteins, in the way that Alex is, or be translated as part of a frameshifted splice variant (e.g., the THPO nirs splice variant, Fig. 6C). Conservation measurements have previously been used to support the functional importance of frameshifted splice variants [24]. Our findings suggest that other conserved frameshifted splice variants, such as caspase 7 isoform beta, are biologically relevant (see Additional file 2).

In a related study by Chung *et al *[8], alternative reading frames (ARFs, which can be considered synonymous with matreshkas) were searched for using a comparative genomics approach in human and mouse. A conservative list of 40 ARF-containing genes was derived from elegant simulation-based statistical and modelling methods. Of these 40 genes, 21 genes were included in our matreshka set (Additional file 4), although the ARF and matreshka sequences were not compared directly, as the former have not been published. Our study had a broader scope, including non-methionine-start reading frames which form the bulk of the longest matreshkas (Table 3). Given the stringent length threshold chosen by Chung *et al*., our analysis probably has a much greater representation of -1 frame matreshkas (see Table 3). All matreshka sequences have been provided as suppplementary data (Additional file 5, Additional file 6), and can be filtered according to our calculated P values (Additional file 3).

## Conclusion

The genome-wide study reported here provides evidence for the existence of potentially dual coding sense-frames in a number of mammalian transcripts. Future studies should aim to determine actual proof-of-translation by raising antibodies against matreshkas, and probing cells or tissues where the mRNAs are known to be expressed. Alternatively genetic mouse models could be generated which would knock-out the putative matreshka while leaving the parent RefSeq sequence intact, thus enabling phenotypic analysis. Future studies could also explore the potential for overlaps between tORFs on the antisense strands. Indeed, genomic mapping of full-length mouse cDNAs has revealed transcriptional forests in which overlap of coding sequences on the sense and antisense strands occurs [25].

## Methods

### Generation of matreshkas

The strategy for identifying conserved frameshifted amino acid sequences (matreshkas) in transcripts is summarised in Fig. 2. First of all, we constructed human-mouse-rat orthologous triplets. Open reading frames (ORFs) were extracted from frames +1 and -1, and translated *in silico*. Sequences above certain conservation thresholds were retained for further analysis.

The starting point for the analysis was the Reference Sequence (RefSeq) set [26]. RefSeq is a well-curated collection of mRNA and corresponding protein sequences maintained by the National Centre for Biotechnology Information (NCBI). Protein entries for human, mouse and rat (release 9) were downloaded from the NCBI web site [10]. Orthologous triplets were built on best reciprocal matches in all vs. all BLASTP searches for human-mouse, human-rat and mouse-rat orthologs. For example, if human protein X is BLASTed against all mouse RefSeq proteins and returns protein Y as best hit, and the converse is true (when protein Y is searched against the human proteome, protein X is the best hit), then proteins X and Y are mutual best matches – and predicted to be *in silico *'orthologs'. If in turn mouse protein Y has rat protein Z as best mutual match, and human protein X had rat protein Z as best mutual match, the ortholog triplet X-Y-Z can be built. 'Best' BLASTP hits were taken as the hits with the highest percentage identity (at least 60%) at the amino acid level to a query, and covering at least 70% of its length. If no hit matching these criteria were found, the ortholog triplet was not built.

The RNA coding sequence for each RefSeq protein in the orthologous sets was then retrieved for *in silico *generation of frameshifted translated ORFs (tORFs). tORFs at least 50 amino acids long were generated computationally from frames +1 and -1 on the sense strand. We used a software package called Scripter developed by ourselves, although this can be done with Getorf from the EMBOSS package [27,28]. Conservation was ascertained with global alignments between all human-mouse and human-rat ORFs within an ortholog triplet. Scripter was again employed for this step, although the EMBOSS package Needle program would be an adequate substitute. Output files were parsed using perl scripts. In a given alignment, the shortest ORF had to be >= 90% the length of the longer, the aligned region had to cover >= 80% of the length of the shorter ORF, and the aligned region had to comprise >= 60% identical amino acid residues. The term 'matreshka' was coined to describe a conserved tORF.

**Figure 8:**
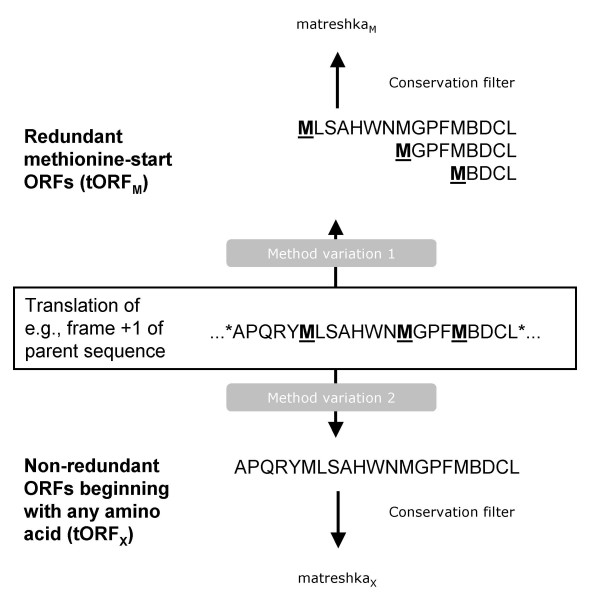
**ORF translation method**. Two variations on the ORF translation step in Fig. 2 were performed. In an effort to identify Alex-like proteins which start with methionine, a first run of the analysis pipeline generated redundant (overlapping) methionine-start ORFs. A second, more comprehensive run generated all ORFs regardless of the first amino acid.

It is important to note that the bioinformatics pipeline was run twice (Fig. 8). The first time ('Method variation 1'), only tORFs beginning with methionine (tORF_M_) were retained, in an effort to identify Alex-like candidates. tORF_M _were allowed to overlap in a redundant fashion (e.g., if a tORF_M _contained two methionines, two tORF_M _would be reported). The second run ('Method variation 2') was more comprehensive, and included all frame +1 and -1 ORF translations (tORF_X_) regardless of the first predicted amino acid (this set was non-redundant). Although the tORF_M _sequences are contained within the tORF_X _set, the subsequent conservation filter produces different matreshka sets.

### Matreshka analysis

A key parameter which differentiated effectively between matreshkas was the number of stop codons which could have arisen to truncate a matreshka, without affecting the 'parent' (frame 0, RefSeq) protein sequence. This is essentially a very approximate measure of the selection pressure maintaining the matreshka. For example, if part of the parent sequence is a valine followed by an isoleucine residue, the possible codons could be GTA, GTT, GTC and GTG (Val) and ATT, ATC and ATA (Ile). It can be seen that there are several codon combinations which could produce a stop codon in a frame +1 matreshka in this part of the sequence (GTGATT, GTGATC, GTGATA, GTAATT, GTAATC and GTAATA). This simple search can be carried out across the length of the parent sequence which overlaps with a matreshka. The final result counts how many times a matreshka could have been truncated by a stop codon without affecting the parent sequence, but was not truncated in either human, mouse or rat.

This stop codon count was supplemented with a probability value which takes codon bias into account, and was calculated as follows. From published codon frequencies [29], we calculated the relative probability of observing each codon for a given amino acid (aa), by dividing the frequency of each codon by the sum of frequencies for all codons for aa 'A'. These relative probabilities must sum to 1 for A. Extending this, we can find the relative probability of observing a di-codon for a pair of amino acids A_1_A_2_. Instead of the binary yes/no answer as to whether a stop codon could truncate a matreshka (described above), we can sum the relative probabilities of di-codons which do not cause a truncation, obtaining a probability that a matreshka is not truncated at a given position, abbreviated here to P_no_stop_. The overall probability of a matreshka 'surviving' is derived by multiplying P_no_stop _across the entire matreshka sequence, abbreviated here to ∏(P_no_stop_). This assumes independence of consecutive (overlapping) bi-codons. We chose to report the probability of a matreshka being truncated by a stop, equal to 1 - ∏(P_no_stop_).

Matreshkas were also scanned for different types of motifs. Those starting with methionine (matreshka_M_, derived from tORF_M_), for instance, might be translated from leaky ribosomal scanning events. A strong Kozak motif could support initiation of translation at these locations [14]. We therefore scanned the nucleotide context around the ATG start for Kozak motifs. The pattern [AG]NNATGG (where N is any nucleotide, and letters in square brackets indicate alternative bases at a given position) was used, derived from published data on initiator codons from highly curated sequences [15]. The significant enrichment of Kozak motifs in the longest matreshka_M _was ascertained with the Exact Binomial Test using R software [30], which was also used for the χ^2 ^test in Table 1. Matreshkas were additionally scanned for signal peptide motifs using SignalP v.2.0 [31]. BLASTP searches of human matreshkas against UniProt (UniRef100, last updated 13^th ^Sep 2005) were carried out with an e-value threshold of 0.0001. To check for repeating sequence elements, the EMBOSS command line programs Equicktandem and Etandem were used with default settings to scan matreshka nucleotide sequences for tandem repeats. SpotFire DecisionSite v. 8.0 (Spotfire Inc., MA, USA), a data visualisation tool, was used to rapidly select the most interesting matreshka candidates.

Matreshka amino acid composition analysis was performed using the EMBOSS program pepstats [28,32]. Sequence annotation, such as the number of introns, flanking genomic sequence and SNP data, was extracted from Ensembl [33,34], and multiple sequence alignments generated with CLUSTAL W v. 1.83 [35,36]. To verify whether the THPO matreshka start is associated with a transcription start site, the program Eponine was used, available from the Sanger Center web site [37].

## Authors' contributions

SR implemented and ran the computational analysis, and drafted the manuscript. AB implemented the ORF extraction and alignment software used, and advised on strategy. BB advised on data analysis and assisted with manuscript preparation. KS advised on strategy and participated in coordination. MRJ conceived and designed the study, and participated in coordination. All authors proofread and approved the final manuscript.

## Supplementary Material

Additional file 1 Bi-codon frequencies Click here for file

Additional file 2 **Frameshifted splice variants**. Human frameshifted PAX8, XBP1 and CASP7 splice variants which contain matreshka sequences. Click here for file

Additional file 3 **Matreshka characteristics**. data on all matreshkas, including sequence identifiers, lengths and number of potential stop codons. Click here for file

Additional file 4 **Matreshka overlap with ARFs**. Table of genes containing alternative reading frames [8] which are represented in the matreshka set. Click here for file

Additional file 5 **Matreshka_M _sequences**. Human matreshka_M _nucleotide sequences in Fasta format. Click here for file

Additional file 6 **Matreshka_X _sequences**. Human matreshka_X _nucleotide sequences in Fasta format. Click here for file

## References

[B1] Sharpless NE, DePinho RA (1999). The INK4A/ARF locus and its two gene products. Current Opinion in Genetics & Development.

[B2] Poulin F, Brueschke A, Sonenberg N (2003). Gene Fusion and Overlapping Reading Frames in the Mammalian Genes for 4E-BP3 and MASK. J Biol Chem.

[B3] Hameed M, Orrell RW, Cobbold M, Goldspink G, Harridge SDR (2003). Expression of IGF-I splice variants in young and old human skeletal muscle after high resistance exercise. J Physiol.

[B4] Klemke M, Kehlenbach RH, Huttner WB (2001). Two overlapping reading frames in a single exon encode interacting proteins – a novel way of gene usage. Embo J.

[B5] Freson K, Jaeken J, Van Helvoirt M, de Zegher F, Wittevrongel C, Thys C, Hoylaerts MF, Vermylen J, Van Geet C (2003). Functional polymorphisms in the paternally expressed XL{alpha}s and its cofactor ALEX decrease their mutual interaction and enhance receptor-mediated cAMP formation. Hum Mol Genet.

[B6] Abramowitz J, Grenet D, Birnbaumer M, Torres HN, Birnbaumer L (2004). XL{alpha}s, the extra-long form of the {alpha}-subunit of the Gs G protein, is significantly longer than suspected, and so is its companion Alex. PNAS.

[B7] Nagy E, Maquat LE (1998). A rule for termination-codon position within intron-containing genes: when nonsense affects RNA abundance. Trends in Biochemical Sciences.

[B8] Chung W-Y, Wadhawan S, Szklarczyk R, Pond SK, Nekrutenko A (2007). A First Look at ARFome: Dual-Coding Genes in Mammalian Genomes. PLoS Computational Biology.

[B9] McCaughan KK, Brown CM, Dalphin ME, Berry MJ, Tate WP (1995). Translational Termination Efficiency in Mammals is Influenced by the Base Following the Stop Codon. Proceedings of the National Academy of Sciences.

[B10] NCBI. http://ncbi.nih.gov/RefSeq.

[B11] Wheeler DL, Barrett T, Benson DA, Bryant SH, Canese K, Chetvernin V, Church DM, DiCuccio M, Edgar R, Federhen S (2007). Database resources of the National Center for Biotechnology Information. Nucl Acids Res.

[B12] Kozmik Z, Kurzbauer R, Dorfler P, Busslinger M (1993). Alternative splicing of Pax-8 gene transcripts is developmentally regulated and generates isoforms with different transactivation properties. Mol Cell Biol.

[B13] Nekrutenko A, He J (2006). Functionality of unspliced XBP1 is required to explain evolution of overlapping reading frames. Trends in Genetics.

[B14] Kozak M (1997). Recognition of AUG and alternative initiator codons is augmented by G in position +4 but is not generally affected by the nucleotides in positions +5 and +6. Embo J.

[B15] Peri S, Pandey A (2001). A reassessment of the translation initiation codon in vertebrates. Trends in Genetics.

[B16] Touriol C, Bornes S, Bonnal S, Audigier S, Prats H, Prats AC, Vagner S (2003). Generation of protein isoform diversity by alternative initiation of translation at non-AUG codons. Biol Cell.

[B17] Matsumoto M, Saito T, Takasaki J, Kamohara M, Sugimoto T, Kobayashi M, Tadokoro M, Matsumoto S-i, Ohishi T, Furuichi K (2000). An Evolutionarily Conserved G-Protein Coupled Receptor Family, SREB, Expressed in the Central Nervous System. Biochemical and Biophysical Research Communications.

[B18] Wu CH, Apweiler R, Bairoch A, Natale DA, Barker WC, Boeckmann B, Ferro S, Gasteiger E, Huang H, Lopez R (2006). The Universal Protein Resource (UniProt): an expanding universe of protein information. Nucl Acids Res.

[B19] Sherry ST, Ward MH, Kholodov M, Baker J, Phan L, Smigielski EM, Sirotkin K (2001). dbSNP: the NCBI database of genetic variation. Nucl Acids Res.

[B20] Jagerschmidt A, Fleury V, Anger-Leroy M, Thomas C, Agnel M, O'Brien DP (1998). Human thrombopoietin structure-function relationships: identification of functionally important residues. Biochem J.

[B21] Park H, Park SS, Jin EH, Song J-S, Ryu S-E, Yu M-H, Hong HJ (1998). Identification of Functionally Important Residues of Human Thrombopoietin. J Biol Chem.

[B22] Pearce KH, Potts BJ, Presta LG, Bald LN, Fendly BM, Wells JA (1997). Mutational Analysis of Thrombopoietin for Identification of Receptor and Neutralizing Antibody Sites. J Biol Chem.

[B23] John MR, Brüngger A, Seuwen K (2003). Identification of Precursor Proteins for Potential Secreted Bioactive Peptides Following a Genome-wide Search [abstract]. J Bone Miner Res.

[B24] Liang H, Landweber LF (2006). A genome-wide study of dual coding regions in human alternatively spliced genes. Genome Res.

[B25] Carninci P, Kasukawa T, Katayama S, Gough J, Frith MC, Maeda N, Oyama R, Ravasi T, Lenhard B, Wells C (2005). The Transcriptional Landscape of the Mammalian Genome. Science.

[B26] Pruitt KD, Tatusova T, Maglott DR (2007). NCBI reference sequences (RefSeq): a curated non-redundant sequence database of genomes, transcripts and proteins. Nucl Acids Res.

[B27] EMBOSS. http://emboss.sourceforge.net.

[B28] Olson SA (2002). EMBOSS opens up sequence analysis. European Molecular Biology Open Software Suite. Brief Bioinform.

[B29] Nakamura Y, Gojobori T, Ikemura T (2000). Codon usage tabulated from international DNA sequence databases: status for the year 2000. Nucl Acids Res.

[B30] Ihaka R, Gentleman R (1996). R: A Language for Data Analysis and Graphics. Journal of Computational and Graphical Statistics.

[B31] Nielsen H, Krogh A (1998). Prediction of signal peptides and signal anchors by a hidden Markov model. Proc Int Conf Intell Syst Mol Biol.

[B32] pepstats. http://www.ebi.ac.uk/emboss/pepinfo.

[B33] Ensembl. http://www.ensembl.org.

[B34] Hubbard TJ, Aken BL, Beal K, Ballester B, Caccamo M, Chen Y, Clarke L, Coates G, Cunningham F, Cutts T (2007). Ensembl 2007. Nucleic Acids Res.

[B35] CLUSTALW. http://www.ebi.ac.uk/clustalw.

[B36] Thompson JD, Higgins DG, Gibson TJ (1994). CLUSTAL W: improving the sensitivity of progressive multiple sequence alignment through sequence weighting, position-specific gap penalties and weight matrix choice. Nucleic Acids Research.

[B37] Down TA, Hubbard TJP (2002). Computational Detection and Location of Transcription Start Sites in Mammalian Genomic DNA. Genome Res.

