# Supplementary material for: Bioinformatics prediction of overlapping frameshifted translation products in mammalian transcripts
Source: BMC Genomics. 2008 Mar 6;9:122. doi: 10.1186/1471-2164-9-122 (PMC2329644; doi:10.1186/1471-2164-9-122)
Supplement: Additional file 2 — Frameshifted splice variants. Human frameshifted PAX8, XBP1 and CASP7 splice variants which contain matreshka sequences. [file 1471-2164-9-122-S2.doc]

**A**

>NP_039246.1 paired box 8 isoform PAX8C [Homo sapiens]

**MPHNSIRSGHGGLNQLGGAFVNGRPLPEVVRQRIVDLAHQGVRPCDISRQLRVSHGCVSKILGRYYETGS**

**IRPGVIGGSKPKVATPKVVEKIGDYKRQNPTMFAWEIRDRLLAEGVCDNDTVPSVSSINRIIRTKVQQPF**

**NLPMDSCVATKSLSPGHTLIPSSAVTPPESPQSDSLGSTYSINGLLGIAQPGSDKRKMDDSDQDSCRLSI**

**DSQSSSSGPRKHLRTDAFSQHHLEPLECPFERQHYPEAYASPSHTKGEQGLYPLPLLNSTLDDGKATLTP**

**SNTPLGRNLSTHQTYPVVA**APPFWICSKSAPGSRPSMPFPMLPPCTGSSRARPSSQGERWWGPRCPDTHP

TSPPADRAAMPPLPSQAWWQEVNTLAMPMATPPTPPTARPGASPTPAC

**B**

>NP_001073007.1 X-box binding protein 1 isoform XBP1(S) [Homo sapiens]

**MVVVAAAPNPADGTPKVLLLSGQPASAAGAPAGQALPLMVPAQRGASPEAASGGLPQARKRQRLTHLSPE**

**EKALRRKLKNRVAAQTARDRKKARMSELEQQVVDLEEENQKLLLENQLLREKTHGLVVENQELRQRLGMD**

**ALVAEEEAEAKGNEVRPVAGSAESAA**GAGPVVTPPEHLPMDSGGIDSSDSESDILLGILDNLDPVMFFKC

PSPEPASLEELPEVYPEGPSSLPASLSLSVGTSSAKLEAINELIRFDHIYTKPLVLEIPSETESQANVVV

KIEEAPLSPSENDHPEFIVSVKEEPVEDDLVPELGISNLLSSSHCPKPSSCLLDAYSDCGYGGSLSPFSD

MSSLLGVNHSWEDTFANELFPQLISV

**C**

>NP_203126.1 caspase 7 isoform beta [Homo sapiens]

**MADDQGCIEEQGVEDSANEDSVDAKPDRSSFVPSLFSKKKKNVTMRSIKTTRDRVPTYQYNMNFEKLGKC**

**IIINNKNFDKVTGMGVRNGTDKDAEALFKCFRSLGFDVIVYNDCSCAKMQDLLKKASEEDHTNAACFACI**

**LLSHGEEN**MESCSVTQAGVQRRDLGRLQPPPPRLAEGPSLMMASRPTRGPSMTQMLILDTRSQWKLTSSS

PIPRFQAITRGGAQEEAPGLCKPSAPSWRSTEKTWKSCRSSPG

## Additional figure 5. Frameshifted splice variants.

A number of matreshka sequences (underlined) match frameshifted splice variants (shown above) from the same gene. Residues in bold are identical in the parental and the frameshifted transcripts. A) a matreshka amino acid sequence generated from PAX8 splice variant A is found in the C variant, B) a matreshka from XBP1(U) matches XBP1(S), C) a matreshka derived from caspase 7 isoform A is present in the beta isoform.
