# Supplementary material for: Bioinformatics prediction of overlapping frameshifted translation products in mammalian transcripts
Source: BMC Genomics. 2008 Mar 6;9:122. doi: 10.1186/1471-2164-9-122 (PMC2329644; doi:10.1186/1471-2164-9-122)
Supplement: Additional file 4 — Matreshka overlap with ARFs. Table of genes containing alternative reading frames [8] which are represented in the matreshka set. [file 1471-2164-9-122-S4.doc]

| **Gene** | **RefSeq Accession** |
| --- | --- |
| SF3A1 | NM_005877 |
| GRP50 | NM_004224 |
| FOXN1 | NM_003593 |
| RXRb | NM_021976 |
| ZNF598 | NM_178167 |
| NOTCH2 | NM_024408 |
| AP3B2 | NM_004644 |
| DLGAP4 | NM_014902 |
| CSRP3 | NM_003476 |
| SEMA6C | NM_030913 |
| LANCL3 | NM_198511 |
| CXXC1 | NM_014593 |
| ADCY8 | NM_001115 |
| SPATA2 | NM_006038 |
| NLGN2 | NM_020795 |
| IQSEC2 | NM_015075 |
| BBX | NM_020235 |
| RBAK | NM_021163 |
| LING01 | NM_032808 |
| KIAA0460 | NM_015203 |
| LPHN1 | NM_001008701 |
